# A New Perspective on Liver Injury by Traditional Chinese Herbs Such As *Polygonum multiflorum*: The Geographical Area of Harvest As an Important Contributory Factor

**DOI:** 10.3389/fphar.2017.00349

**Published:** 2017-06-20

**Authors:** Longfei Lin, Hui Li, Hongmei Lin, Miao Zhang, Changhai Qu, Lei Yan, Xingbin Yin, Jian Ni

**Affiliations:** ^1^Institute of Chinese Materia Medica, China Academy of Chinese Medical SciencesBeijing, China; ^2^School of Chinese Materia Medica, Beijing University of Chinese MedicineBeijing, China; ^3^Fengtai District Community Health CenterBeijing, China

**Keywords:** *Polygonum multiflorum*, toxicity, geographical areas, PCA, UPLC-Q-TOF/MS

## Abstract

Herbal medicine has been widely used in the treatment of various diseases; however, the adverse reactions cannot be ignored. Most previous studies have ignored the relationship between the factors of geographical areas/batches and toxicity. This study used *Polygonum multiflorum* (PM) as an example to analyze the relationship between the geographical areas/batches and toxicity and speculated on the hepatotoxicity-inducing components in PM based on high content screening, UHPLC-Q-TOF/MS and Progenesis QI software analysis. The results of the study show that the toxicity of PM was obviously different among the different geographical areas, and the most toxic PM was from the Sichuan province. To obtain more accurate results and to reduce the false-positive rate, two methods were used to evaluate the speculative results. It was noteworthy that emodin was not the main hepatocyte toxicity constituent of PM. The analysis methods suggested that PM toxicity may be associated with tetrahydroxystilbene-*O*-(galloyl)-hex and emodin-*O*-hex-sulfate. The toxicity of these two components requires further study.

## Introduction

Currently, there are many reports on the adverse reactions of Chinese herbal medicines (CHM) including *Polygonum multiflorum* (PM) (Park et al., 2001; Dong et al., 2014), rhubarb (Wang et al., 2011; Albersmeyer et al., 2012), *Tripterygium wilfordii* (Li et al., 2015), *Fructus Aristolochiae* (Abdelgadir et al., 2011; Yuan et al., 2014; Wu et al., 2016), *Radix aconiti carmichaeli* (Murayama et al., 1991; Zhang et al., 2016), *Periploca sepium bunge, Nuces vomicae* (Ng et al., 2013) and others. Some of these toxic components are unambiguous; for example, aristolochic acid, which is present in *fructus aristolochi*, has renal toxicity (Mengs and Stotzem, 1993; Okada et al., 2003), and the cardiac glycosides in *P. sepium bunge* can induce arrhythmia (Demek and Sporni, 1969; Kobinger et al., 1970). In general, these studies have ignored the relationship between the factors of geographical areas/batches and toxicity. However, the batch and product variability of herbs is an important factor for herbal hepatotoxicity assessments (Teschke et al., 2013). The Adverse Drug Reaction (ADR) monitoring system in China also considers the place of origin of the herbs as an important factor in their adverse reactions (Zhang et al., 2012). Furthermore, some CHM ingredients that induce adverse reactions, such as PM, are ambiguous.

**FIGURE 1:**
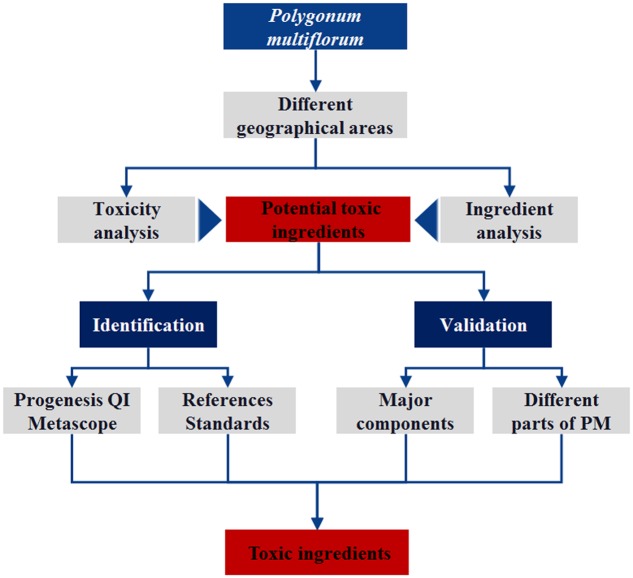
Schematic diagram of the experimental process.

*Polygonum multiflorum* consists of the roots of the *Polygonaceae* plant *Radix polygoni multiflori*. PM can be used in the treatment of hypertension (Ding and Zhu, 2009), NAFLD (Yang et al., 2013), Alzheimer’s disease (Chen et al., 2010) and other clinical diseases (Zhu, 2011; Lin et al., 2015b). Currently, clinical reports on adverse reactions resulting from PM and its preparations, especially liver injury, have received wide attention. The Medicines and Healthcare Products Regulatory Agency has issued relevant information about the adverse reactions of PM. Currently, liver injuries (especially acute injuries) caused by taking PM have been reported worldwide (But et al., 1996; Mazzanti et al., 2004; Panis et al., 2005). These include 66 herb-induced liver injury (HILI) cases reported by 302 Military Hospital in China, which were associated with PM and preparations of its compounds (Zhu et al., 2016) using the Roussel Uclaf Causality Assessment Method (RUCAM) (Danan and Teschke, 2016). In the present study, PM was used as an example to analyze the relationship between the geographical areas/batches and toxicity and to speculate on the hepatotoxicity-inducing components in PM. Although the analytical procedure is consistent with the method we have previously used (Lin et al., 2015a), more attention was paid to the verification of the speculative results to reduce the false-positive results in this study. The experimental procedure is shown in **Figure 1**.

## Materials and Methods

### Chemicals

Methanol (HPLC-grade) was purchased from Fisher (United States). A Cascada^TM^ IX-water Purification System (Pall Co., United States) was used to provide high purity water. The standard for emodin, 2,3,5,4′-tetrahydroxystilbene-2-O-β-glucoside (TSG) was provided by Shanghai Standard Biotech Co., Ltd (Shanghai, China). Emodin-8-O-β-D-glucopyranoside (EDG) was provided by Shanghai Yuanye Bio-Technology Co., Ltd. (Shanghai, China). The different geographical areas/batches of PM samples (21 batches) were purchased from Anguo medicine market. Dulbecco’s modified Eagle’s medium (DMEM), penicillin, streptomycin, and fetal bovine serum (FBS) were obtained from Gibco-Life Technologies (United States). A Cell Counting Kit (CCK-8) was obtained from Dojindo (Tokyo, Japan).

### Preparation of PM Samples from Different Geographical Areas/Batches and Constituent Analysis

The powdered samples (4.0 g crushed and passed through a No. 4 sieve) were extracted with 20 volumes of methanol for 60 min, after which the supernatants were cooled to room temperature, filtered and evaporated to dryness. The residue was dissolved in 5 mL of 50% dimethyl sulfoxide (DMSO–water solution). The DMSO solutions were diluted with 10% FBS (HyClone, Logan, UT, United States) and antibiotics (100 U/mL penicillin and 100 μg/mL streptomycin) in DMEM (DMEM, Gibco Invitrogen Corp., Grand Island, NY, United States) to the concentrations shown in **Table 1**. To facilitate the comparison of the toxicity of PM among the various geographical areas/batches, the concentrations of the samples that were used for the cell counting kit (CCK-8) reduction assay were calculated on the quantity of crude drug/mL.

**Table 1:** Cell Counting Kit-8 assay results and IC_50_ for 21 batches of *Polygonum multiflorum* (means ± SD).

Batch NO.	Concentration (mg *crude drug*/mL)						IC_50_
	2	1	0.5	0.25	0.125	0.0625	
1	14.34 ± 0.06	20.46 ± 0.49	48.84 ± 2.16	79.89 ± 2.92	95.67 ± 0.88	99.1 ± 1.18	0.83
2	13.05 ± 0.42	15.21 ± 0.59	40.62 ± 0.67	79.63 ± 5.70	88.32 ± 2.15	95.15 ± 2.65	0.757
3	14.33 ± 2.42	14.64 ± 0.31	32.01 ± 1.56	73.14 ± 1.65	95.44 ± 3.34	99.81 ± 9.37	0.715
4	13.32 ± 0.92	17.62 ± 0.15	32.48 ± 1.95	60.4 ± 1.58	84.14 ± 1.14	98.53 ± 7.74	0.687
5	14.5 ± 0.33	16.1 ± 0.61	44.61 ± 2.04	73.85 ± 3.61	92.04 ± 6.45	95.35 ± 6.52	0.779
6	11.4 ± 2.08	15.08 ± 2.66	39.76 ± 2.14	69.53 ± 2.52	86.1 ± 3.56	94.36 ± 4.92	0.692
7	7.11 ± 0.42	41.2 ± 0.49	72.7 ± 2.28	86.2 ± 2.77	90.81 ± 2.34	93.58 ± 1.14	0.944
8	14.72 ± 0.32	54.74 ± 1.12	78.76 ± 4.42	86.69 ± 1.43	92.37 ± 2.86	93.15 ± 1.63	1.151
9	41.81 ± 1.25	74.14 ± 2.62	85.8 ± 2.74	88.12 ± 0.66	96.55 ± 6.47	98.87 ± 4.50	1.73
10	8.8 ± 0.60	59.68 ± 0.91	89.83 ± 0.43	91.27 ± 3.22	96.19 ± 7.60	98.45 ± 0.31	1.177
11	18.15 ± 3.69	70.09 ± 4.47	90.46 ± 3.62	97.28 ± 10.64	102.04 ± 7.52	102.41 ± 9.82	1.383
12	36.44 ± 1.56	71.8 ± 7.75	87.57 ± 8.66	93.19 ± 12.92	98.18 ± 7.86	101.24 ± 1.40	1.629
13	34.75 ± 2.43	75.57 ± 2.31	94.37 ± 1.55	97.56 ± 2.96	97.32 ± 1.85	101.17 ± 2.85	1.656
14	17.17 ± 2.24	51.62 ± 1.88	80.41 ± 3.36	90.95 ± 5.74	94.75 ± 4.52	98.98 ± 4.76	1.199
15	34.62 ± 4.39	70.94 ± 10.66	87.8 ± 5.36	93.14 ± 6.65	96.17 ± 11.97	100.06 ± 4.16	1.595
16	32.64 ± 3.44	67.14 ± 2.48	85.43 ± 6.43	93.94 ± 7.34	95.51 ± 13.55	98.95 ± 8.65	1.527
17	16.65 ± 0.52	51.36 ± 3.03	86.82 ± 2.35	92.07 ± 3.44	95.24 ± 6.59	96.27 ± 3.72	1.213
18	17.27 ± 2.33	54.25 ± 2.58	83.96 ± 1.94	88.16 ± 6.78	88.92 ± 1.83	90.44 ± 1.16	1.195
19	26.83 ± 1.45	72.48 ± 3.09	93.47 ± 5.56	94.69 ± 3.25	101.31 ± 4.30	101.2 ± 12.36	1.515
20	30.74 ± 1.42	67.48 ± 7.03	87.54 ± 3.40	93.71 ± 2.37	96.61 ± 3.79	100.36 ± 8.12	1.382
21	31.3 ± 2.39	71.67 ± 3.19	88.91 ± 5.47	98 ± 3.84	99.35 ± 2.90	99.95 ± 1.29	1.562

The stock solutions in DMSO that were used for the cell assays were diluted 100-fold with a 50% methanol–water solution, vortexed for 30 s and then centrifuged at 14,000 rpm for 10 min. Then, the supernatants were centrifuged again at 14,000 rpm for 10 min and collected for UPLC-Q-TOF/MS analysis. The analysis of the main constituents of the various fractions in the PM from various sources was performed on a Waters Xevo G2 Q-TOF/MS (Waters Corp., Milford, MA, United States) using the negative detection mode. The mass spectrometric parameters were as follows: Cone gas flow rate: 50 L/h; Source temperature: 120°C; Capillary: 2.5 kV; Desolvation temperature: 350°C; Gas flow rate: 600 L/h; Full-scan range: 50 to 1200 m/z.

### The Cytotoxicity and Hepatotoxic Assay

#### Culture Conditions

L02 cells (also called HL-7702 cells), a normal human liver cell line, were purchased from the China Infrastructure of Cell Line Resources. The L02 cells were cultured in DMEM supplemented with 10% FBS in a humidified incubator with 5% CO_2_ and 95% air at 37°C. The cells were passaged at 80–90% confluence using trypsin (0.5%, Sigma, St. Louis, MO, United States) digestion.

#### Cell Viability Assay (CCK-8)

The CCK-8 reduction assay, which is based on WST-8 ([2-(2-methoxy-4-nitrophenyl)-3-(4-nitrophenyl)-5 -(2,4-disulfophenyl)-2H-tetrazolium), was used to determine the cell proliferation and cytotoxicity. WST-8 is reduced by dehydrogenases in the cells to give a yellow-colored product (formazan), which is soluble in the tissue culture medium. The amount of the formazan dye generated by the activity of dehydrogenases in cells is directly proportional to the number of living cells. The L02 cells were plated at a density of 5 × 10^3^ cells/well in 96-well plates (Corning, NY, United States) and incubated for 24 h. The cells were exposed to a fresh medium that contained various concentrations of the extracts for 36 h beginning after the 24 h incubation period following plating. After the incubation with the extracts, the medium was removed, and 100 μL of fresh DMEM containing 10 μL of the CCK-8 solution was added to each well of the 96-well plates, which were then incubated for 3.5 h. The absorbance of the samples was measured at 450 nm using a microplate reader (Thermo, Multiskan, GO, United States).

#### The Hepatotoxicity Assay by High Content Screening

High content screening (HCS) is used to identify the hepatotoxicity of compounds and extracts by simultaneous detection of cell loss, nuclear DNA, reduced glutathione (GSH) level, intracellular reactive oxygen species (ROS) and mitochondrial membrane potential (MMP) as multiplexed targets of hepatotoxicity. The L02 cells were seeded into collagen-I-coated clear-bottom 96-well plates (5 × 10^3^ cells/well, BD BioCoat^TM^ Plate Product) and incubated for 24 h. The cells were exposed to various concentrations of the extracts and control drugs (negative: aspirin; positive: ticlopidine, which were formulated according to the instructions of the drug-induced liver injury assay cartridge) for 36 h. Following this incubation, the medium was then removed and 100 μL well of the dye mixture, which contained Hoechst 33342, mBCl dye, ROS dye and Mito dye, was added to the 96-well plates, and the plates were incubated for 45 min. The absorbance changes of the specimens were measured using Thermo Scientific Cellomics ToxInsight (Thermo Scientific Cellomics, United States).

### Data Statistics and Analysis

The cytotoxicity results were expressed as the means ± S.D., which were calculated from the triplicate experiments. SPSS 17.0 was used to calculate the IC_50_ of each tested compound or extract. The cytotoxicity of the various areas/batches PM was difficult to distinguish using only the IC_50_ or inhibition rate at one concentration. In the experiment, hierarchical cluster analysis (HCA) and principal component analysis (PCA) were used to identify and distinguish the relatively homogeneous groups of cases based on the IC_50_ and the inhibition rates on all concentrations. The fingerprint of the chromatographic peaks from the UHPLC-Q-TOF/MS analysis used Progenesis QI and Ezinfo software for PCA and OPLS-DA analysis.

## Results and Discussion

### The Cytotoxicity of PM from Various Geographical Areas and Batches

**FIGURE 2:**
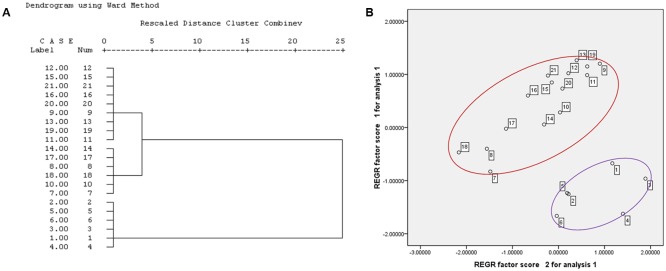
Hierarchical cluster analysis and PCA results for the cytotoxicity of 21 batches PM **(A)** HCA result; **(B)** PCA result.

The changes in cell proliferation and the IC_50_ after exposure to 21 batches of PM extracts are listed in **Table 1**. The results showed that all 21 batches of the PM samples inhibited the cell proliferation, and the variations of the cytotoxicity were obvious in the samples from different geographical areas or even different batches from the same geographical areas. For example, the IC_50_ values for batches 1–7 were less than 1.0 mg crude drug/mL whereas the others were greater than this concentration. To evaluate the differences in the toxicity of various geographical areas/batches of PM, HCA was performed on the basis of the characteristics of the different toxicity evaluation indices. The toxic index in the 21 batches of PM samples formed a 21 × 7 matrix. The distances between the 21 samples were calculated using the SPSS software. As shown in **Figure 2A**, samples 1–6 were from the Sichuan province, samples 7–12 were from the Hunan province, samples 13–18 were from the Guzhou province, and samples 19–21 were from the Guangdong province. The HCA clearly indicated that the toxicity of PM from different provinces, or even different batches from the same province, were significantly different. The HCA results indicated that the toxicity of 21 batches of PM was divided into two regions, and samples 1–6 were more potent than the other 15 batches. **Figure 2B** shows the scores of the different toxicity evaluation indices for the 21 batches of PM after the dimensions were reduced by PCA. The scatter was also divided into two regions in which a discrete degree of scatter combined with good clustering was observed in the PCA scores. This result was consistent with the results of the HCA, which indicated that the PM samples from the Sichuan province (batches 1–6) were more toxic than those from the other provinces (batches 7–21), and the differences in the toxicity were due to the differences in the composition.

### Prediction of the Potentially Toxic Constituents in PM

**FIGURE 3:**
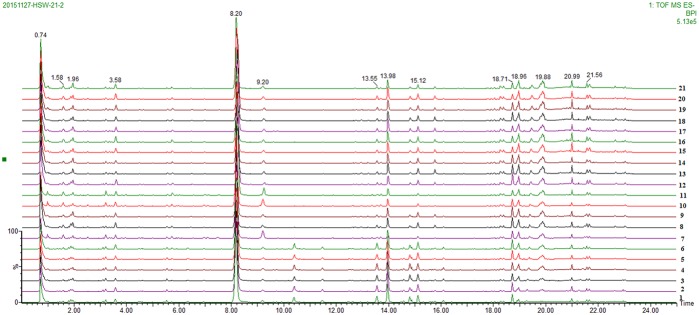
The base peak ion chromatogram for 21 batches of PM.

A total of 21 batches of PM were analyzed using UPLC-QTOF/MS (three replicate samples for each batch), and the base peak ion chromatogram is shown in **Figure 3**. In this study, the analytical procedure was mainly performed using the Progenesis QI software, which is consistent with the analytical method we have previously used.

**FIGURE 4:**
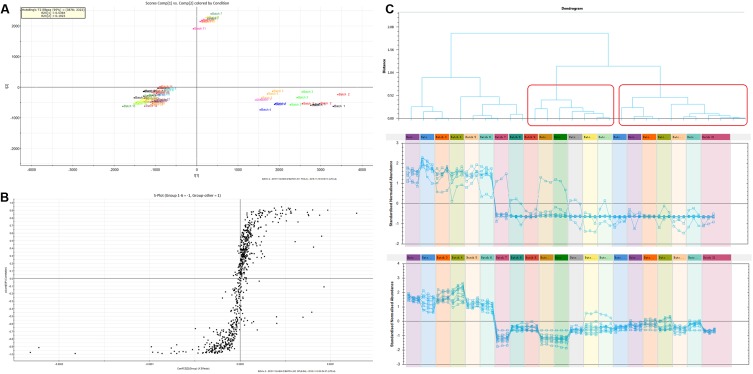
**(A)** The PCA scores of the component difference among the 21 batches; **(B)** OPLS-DA scatter plots of the difference between batches 1–6 and batches 7–21; **(C)** The results of toxic components predicted by the correlation analysis.

First, the TIC spectra of all samples that were collected using UPLC-Q-TOF/MS were translated into a 2D ion intensity map (Horizontal: retention time; vertical coordinates: *m/z*) using the Progenesis QI software. Then, the alignment and peak-picking procedures were run. The peaks that were observed by peak-picking were exported into EZinfo (PCA analysis software) for the next step of the analysis. Second, the outliers and classification trends among these extracts were evaluated using unsupervised PCA. Three clusters were observed in the score plot, which clearly differentiated among these extracts. Batches 1–6 clustered together, batches 7, 10 and 11 were another cluster, and the other batches (11–21) composed the last cluster, as shown in **Figure 4A**. It was exciting that the results of the component differences among the 21 batches of PM were consistent with the toxicity results. Therefore, the component differences between batches 1–6 and batches 7–21 may be the reason for the difference in toxicity. The component differences between batches 1–6 and batches 7–21 were obtained using scatter plots generated using OPLS-DA as shown in **Figure 4B**. The characteristic components with the highest credibility between groups (which were also the significantly different variables) were located at both ends of the S-curve and displayed a large absolute value of p(corr)[1] and coefficients. A total of 28 compounds were found to differ between batches 1–6 and batches 7–21. Correlation analysis was adopted for these compounds through Progenesis QI software as shown in **Figure 4C**. A description of these constituents is shown in **Table 2**. The results showed that 16 compounds were abundant in batches 1–6 but were low in the other batches of PM. These constituents may be the reason that the toxicity of the PM provided by the Sichuan province was greater than that of the samples from the other geographical areas. The identification procedure for the 16 potentially toxic constituents is provided in the “Supplementary Material.”

**Table 2:** The potentially toxic compounds predicted by correlation analysis and peak areas in the various fractions of *Polygonum multiflorum.*

NO.	m/z	*t*_R_	p[1]P	p(corr)[1]P	Factor of Change(Batch1-6 vs. Batch7-21)	Identification
1	577.1347	3.09	-0.0673	-0.88966	2.3	Procyanidin B2
2	729.1462	6.34	-0.0995	-0.981456	220.8	Galloyl-procyanidin
3	405.1186	8.2	-0.3917	-0.637517	1.2	TSG
4	557.1295	10.39	-0.2456	-0.986749	76.7	Tetrahydroxystilbene-O-(galloyl)-hex
5	431.0973	11.46	-0.1249	-0.991541	7.9	Emodin-O-glc
6	511.0542	12.49	-0.0929	-0.954889	165.6	Emodin-O-hex-sulfate
7	407.1335	13.55	-0.1339	-0.961387	2.8	Torachrysone-glucoside
8	431.0975	13.98	-0.363	-0.981783	2.6	EDG
9	583.1088	14.47	-0.0666	-0.9842	153.2	Unknown
10	559.1449	14.58	-0.1004	-0.992625	10000	Torachrysone-O-glucogallin
11	473.108	14.81	-0.1589	-0.871941	2.9	Emodin-O-(acetyl)-hex
12	517.098	14.81	-0.1094	-0.903225	3.7	Emodin-O-(malonyl)-hex
13	449.1442	14.88	-0.0945	-0.954155	4.4	Pinostilbenoside
14	283.06	15.12	-0.135	-0.82364	1.8	Anthraquinone
15	445.1148	15.12	-0.0917	-0.970935	4.6	Glucoobtusifolin
16	283.06	16.1	-0.0857	-0.914172	3	Anthraquinone

### Validation of Potential Toxic Constituents

The speculative results assumed that the analysis results of this research are similar to previous studies. However, there are some differences, such as the fact that emodin was not present in this speculative result. It is not clear whether emodin has no toxic effect or the toxicity of emodin has a small contribution to the toxicity of PM. This also indicates that there are some false-positive results in the speculated results. To obtain more accurate results, two methods were adopted to verify their accuracy.

(I) The first method evaluated the cytotoxicity and hepatotoxicity of the existing compounds (including TSG, EDG and emodin) in PM through CCK-8 assay and HCS to validate the speculated results.
(II) The second method was to separate the PM into different extracted fractions according to the type of constituents in PM, including the **total**, the **dichloromethane (DCM) fraction** (the content of which was mainly free anthraquinone), the **water fraction** (which consisted mainly of tannins and polysaccharides), the **30% ethanol fraction** (mainly composed of polyhydroxystilbenes such as TSG) and the **70% ethanol fraction** (mainly composed of conjugated anthraquinones such as EDG). The extraction and separation procedures and the results of the analysis of the main components of these five extract fractions are provided in the “Supplementary Material.” Finally, the speculative results were validated by investigating the cytotoxicity and hepatotoxicity of these extracts.

#### The Cytotoxic of Different Fractions of PM and Its Three Major Components Using the CCK-8 Assay

To evaluate the toxicity of different fractions of PM and three of its major components, the CCK-8 assay was used to determine cell viability before performing HCS *in vitro*. The changes in cell proliferation after exposure to the total fraction, the water fraction, the 30% ethanol fraction, the 70% ethanol fraction and the DCM fraction are listed in **Table 3**. The total fraction showed significant cytotoxicity, with an 83.56% inhibition of the cell growth at 1100 μg/mL. The cytotoxicity of this extract was the highest of the five fractions tested. The cytotoxicity of the 30% ethanol fraction was the second most toxic as indicated by the 71.54% inhibition of the cell growth at 220 μg/mL. The cytotoxicity of the 70% ethanol fraction was weaker than the 30% ethanol fraction. The cell survival rates after treatment with the 70% ethanol fraction were higher than the rates for treatment with the 30% ethanol fraction in the CCK-8 assay, with 26.33% inhibition at 70 μg/mL. The toxicities of the water and DCM fractions were the weakest of these five extracts, as indicated by markedly lower inhibition of the growth.

**Table 3:** Cell Counting Kit-8 assay results for the various fractions of *Polygonum multiflorum*.

mg *crude drug*/mL	Total		Water		30 Ethanol		70 Ethanol		DCM	
	μg/mL	Cell proliferation (%)	μg/mL	Cell proliferation (%)	μg/mL	Cell proliferation (%)	μg/mL	Cell proliferation (%)	μg/mL	Cell proliferation (%)
3	1100	16.44 ± 0.35	720	85.25 ± 1.39	220	28.46 ± 1.10	70	73.67 ± 3.98	16	82.52 ± 0.87
1.5	550	42.24 ± 1.04	360	88.13 ± 1.27	110	53.95 ± 1.93	35	78.85 ± 2.91	8	88.95 ± 0.32
0.75	275	73.95 ± 1.81	180	92.37 ± 1.82	55	86.64 ± 1.97	17.5	82.89 ± 4.68	4	93.34 ± 4.22
0.375	137.5	89.94 ± 1.73	90	93.11 ± 1.28	27.5	89.83 ± 4.46	8.75	86.77 ± 6.92	2	93.75 ± 2.20
0.1875	68.75	96.83 ± 2.85	45	94.72 ± 1.16	13.75	92.42 ± 3.60	4.38	88.56 ± 5.63	1	95.62 ± 4.33
0.09375	34.38	100.51 ± 1.22	22.5	94.79 ± 2.90	6.88	92.13 ± 5.30	2.19	94.13 ± 3.59	0.5	97.31 ± 3.98

The cytotoxicity of emodin, which inhibited cell growth 71.54% at 220 μg/mL, was stronger than that of TSG and EDG. TSG and EDG did not obviously inhibit the cell proliferation at a concentration of 100 μg/mL. The changes in cell proliferation are listed in **Table 4**.

**Table 4:** Cell Counting Kit-8 assay results for three major constituents of *Polygonum multiflorum*.

μg/mL	TSG	Emodin	EDG
	Cell proliferation (%)	Cell proliferation (%)	Cell proliferation (%)
100	97.43 ± 2.38	23.12 ± 1.14	78.65 ± 2.69
50	101.27 ± 2.02	44 ± 1.79	81.93 ± 0.33
25	101.03 ± 1.98	55.66 ± 1.02	84.63 ± 2.83
12.5	100.48 ± 0.89	76.3 ± 1.42	92.06 ± 0.87
6.25	100.96 ± 2.56	81.74 ± 0.64	92.94 ± 4.99
3.125	99.73 ± 1.68	94.33 ± 4.53	97.54 ± 2.6

#### The Hepatotoxicity of the Various Fractions of PM and Its Three Major Components by HCS

**FIGURE 5:**
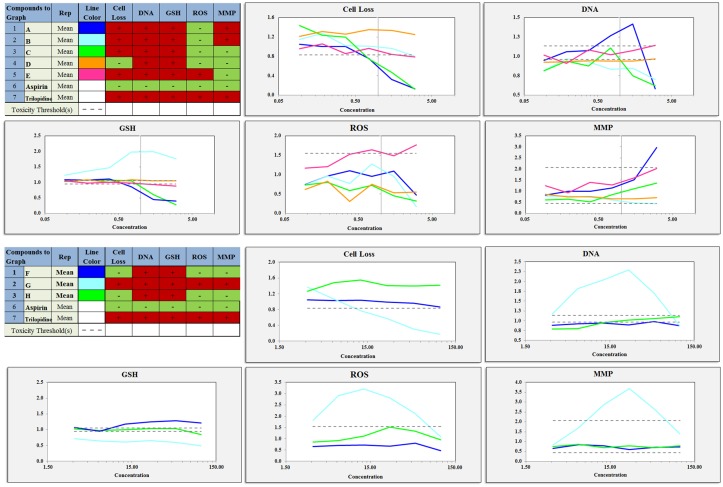
Various concentrations of the components and extracts inhibited cell proliferation, DNA damage, GSH levels, ROS levels, mitochondrial membrane potential changes in proliferating cells. A, total extract; B, Water fraction; C, 30% ethanol fraction; D, 70% ethanol fraction; E, DCM fraction; F, TSG; G, EDG; H, Emodin.

The HCS results for various concentrations of the five extracts and the two control compounds (negative: aspirin; positive: ticlopidine) are shown in **Figure 5**. The five indexes were positive for ticlopidine and negative for aspirin. These results showed that the positive drug was obviously toxic to the L02 cells whereas aspirin was non-toxic.

**FIGURE 6:**
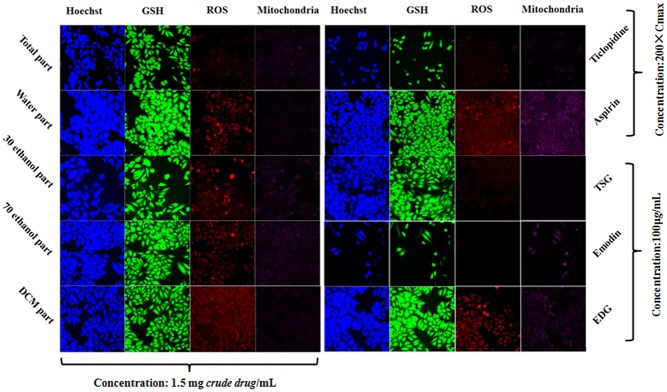
Comparison of the components and extracts on the cell state and fluorescence intensity in L-02 cells.

The cell loss induced by the total extract and the 30% ethanol fraction were more severe than that induced by the other fractions. The DCM fraction and the water fraction also showed a downward trend and decreased to below the toxicity threshold. This result was similar to that of the CCK-8 assay. The Hoechst 33342 staining results showed that the nuclei of the cells in the aspirin group were homogeneously fluorescent, whereas nuclear shrinkage, chromatin condensation, a significantly reduced cell number, and cell apoptosis were observed for the other fractions and ticlopidine groups, as shown in **Figures 5**, **6**. The GSH levels of the total extract, the 30% ethanol fraction, the 70% ethanol fraction and the DCM fraction decreased as the dose increased: the effects of total extract and the 30% ethanol fraction were more obvious compared with the other groups. However, the GSH levels increased following exposure to the water fraction, and at all concentrations, these levels remained above the threshold. ROS, which have the potential to cause damage to cellular DNA, RNA and proteins, were produced. The levels of ROS increased as a function of the dose of the DCM fraction. A normal MMP is a prerequisite for maintaining oxidative phosphorylation, which produces ATP in the mitochondria, and a stable MMP is needed to maintain the normal physiological function of cells. The high content fluorescence imaging system results showed that the level of MMP increased significantly with increasing concentrations of the total extract and ticlopidine, and exceeded the safety threshold as defined by the negative control group.

The HCS result showed that emodin caused changes in the cell loss, nuclear DNA, GSH, ROS and MMP levels, and it also indicated that emodin is an important chemical constituent that induces liver cell damage. This result was consistent with the literature (Qu et al., 2013). However, whether emodin was the main component caused liver cell damage in PM, it will be made a detailed analysis in next section. Although there were changes in the DNA and GSH levels following exposure to TSG and EDG, there were no significant changes in the number of cells. This also suggests that high concentrations of TSG and EDG might lead to liver cell damage. These results are also shown in **Figures 5**, **6**.

#### Speculative Result Verification by the Combination of Two Methods

The cytotoxicity and hepatotoxicity results indicated that emodin was more toxic than TSG and EDG, and the toxicity of total extract was greater than that of the other fractions. However, the concentration of emodin was only 2.45 μg/mL when the concentration of the total extract was 1100 μg/mL. According to the CCK-8 and HCS assay results, emodin did not inhibit the cell proliferation or cause hepatotoxicity at a concentration of 2.45 μg/mL. These results indicated that emodin was not the major hepatotoxic constituent of PM. Meanwhile, the DCM fraction, which mainly contains free anthraquinones, also did not strongly inhibit the cell proliferation. These results also indicated that the free anthraquinones (including emodin) and pinostilbenoside were not the major hepatocyte toxicity constituents of PM.

Similarly, although EDG could lead to liver cell damage at a concentration of 100 μg/mL, the concentration of EDG was only 11 μg/mL when the concentration of the total extract was 1100 μg/mL. The 70% ethanol fraction, in which the content was mainly a conjugated anthraquinones, also did not strongly inhibit the cell proliferation. These results indicated that the sum of the combined anthraquinones (such as EDG) was not the primary constituent that causes hepatotoxicity in PM.

In this study, the 30% ethanol fraction (which mainly consisted of TSG) was strongly cytotoxic, and the cell loss, nuclear DNA, GSH, ROS and MMP levels of L-02 cells induced by the 30% ethanol fraction were similar to those of the total extract based on the CCK-8 and HCS assays. The water fraction, the 70% ethanol fraction and the DCM fraction were weakly cytotoxic and hepatotoxic compared with the 30% ethanol fraction in the same samples of the crude herbs. Some components in the 30% ethanol fraction of PM had a strong toxic effect, suggesting that these constituents were the main source of hepatotoxicity induced by PM. The total extract and the 30% ethanol fraction inhibited the cell growth by 83.56% at 1100 μg/mL and 71.54% at 220 μg/mL, respectively, which indicated significant cytotoxicity and hepatotoxicity. According to the results of the TSG content determination, this compound is mainly present in the 30% ethanol fraction. The concentration of TSG was 105 μg/mL when the concentration of the total extract was 1100 and 78 μg/mL when the concentration of the 30% ethanol fraction was 1100 μg/mL. However, the CCK-8 and HCS assays indicated that the TSG monomer did not show strong cytotoxicity and hepatotoxicity at a concentration of 100 μg/mL. Therefore, TSG was also a false-positive result, and there were other constituents of the 30% ethanol fraction that are the main toxic component in PM.

The correlation analysis results of toxicity and the relative content of 16 potential toxic constituents of the various fractions of PM using UHPLC-Q-TOF/MS and the Progenesis QI software showed that the relative content of tetrahydroxystilbene-*O*-(galloyl)-hex TSG and emodin-*O*-hex-sulfate were positively correlated with toxicity of different fractions of PM, and the content of these compounds were also high in the total extract and 30% ethanol fraction. The analysis results are provided in the “Supplementary Material.” Other constituents such as EDG, emodin-O-glc, torachrysone-glucoside, galloyl-procyanidin, etc., might be false-positive results.

The above results illustrate that, although emodin has a strong toxic effect (Wang et al., 2007), it might not be the main constituent that causes hepatocyte toxicity in PM due to its low content. TSG was a false-positive result, as indicated by its weak toxic effect despite its high content in PM. EDG, pinostilbenoside, emodin-*O*-glc, torachrysone-glucoside and free anthraquinone also showed false-positive results. Combined with correlation analysis results of the content of 16 potential toxic constituents in the various fractions of PM, only two components, tetrahydroxystilbene-*O*-(galloyl)-hex and emodin-*O*-hex-sulfate, might be the main toxic components in PM.

## Conclusion

*Polygonum multiflorum* is a widely used herbal medicine in China and Japan, and there were 61 Chinese patent drugs collected in *Chinese Pharmacopoeia 2015 edition* that contained PM or its preparations. The reports and research on the hepatotoxicity of PM and its preparations have increased (Cho et al., 2009). Some studies have suggested that PM-induced liver injury is idiosyncratic and have established a relevant animal model (Fan et al., 2015; Li C. Y. et al., 2016). However, other studies have also shown that PM induced liver injury with dose-toxicity relationship (Ma et al., 2015; Li H. et al., 2016), and some reports on the internet have also indicated that excessive consumption of PM can cause liver failure. The China Food and Drug Administration (CFDA) has limited the dose of raw PM to 1.5 g/day and processed PM to 3.0 g/day and added subjects with “Liver dysfunction, family history of liver disease” as unsuitable for the use of healthy food that contains PM. The CFDA has also encouraged research on the mechanism of the liver injury caused by PM, including identifying diagnostic biomarkers for early warning and detection of the liver injury induced by PM in clinical studies (Teschke et al., 2016).

However, the hepatotoxic components of PM that result in liver injury have not been clearly shown despite the wide concerns in recent years aroused by reports of PM-induced hepatotoxicity. Some studies considered the toxic constituent to be emodin whereas some considered it to be the combined anthraquinones. Tannins, TSG and other components have also been considered (Yu et al., 2011; Lv et al., 2015; Li et al., 2017; Wang et al., 2017). There were substantial differences in the contents of the constituents among the different geographical areas/batches of PM (Lin et al., 2015c). The toxicity of PM obtained from different geographical areas or batches should, therefore, be investigated. The results of our study show that the toxicity of PM was clearly different among the different geographical areas: in particular, the most toxic PM was from the Sichuan province. The differences in the composition of the PM obtained from the Sichuan province compared to those from the other provinces were analyzed by Progenesis QI software and UHPLC-Q-TOF/MS. To obtain more accurate results and reduce the false-positive rate, two methods were used to verify the speculative results. It was noteworthy that emodin was not the main toxic constituent that caused the hepatocyte toxicity of PM, and the toxicity of PM may be related to tetrahydroxystilbene-*O*-(galloyl)-hex and emodin-*O*-hex-sulfate. Further studies are warranted to investigate the toxicity of these two components. The studies also should pay attention to improving the processing technology to decrease the content of these two components of PM, which could reduce the liver toxicity and ensure the safety of clinical applications. Furthermore, the quality of CHM should be strictly controlled, and evidence-based clinical trials should be performed using scientific methods such as RUCAM to assess causality for herbs in suspected liver injury (Danan and Teschke, 2016). Then, a positive risk/benefit profile should be confirmed to enhance its globalization (Teschke et al., 2014; Teschke and Eickhoff, 2015).

## Author Contributions

LL and HuL conducted the main experiments; LY determined the biochemical and antioxidant indices; LL, LY, and MZ wrote the manuscript and prepared the figures; CQ and JN performed the data analysis; HoL and XY revised the manuscript; JN conceived the study. All authors reviewed the manuscript.

## Conflict of Interest Statement

The authors declare that the research was conducted in the absence of any commercial or financial relationships that could be construed as a potential conflict of interest.

## References

[B1] Abdelgadir A. A. Ahmed E. M. Eltohami M. S. (2011). Isolation, characterization and quantity determination of aristolochic acids, toxic compounds in *Aristolochia bracteolata* L. *Environ Health Insights* 5 1–8. 10.4137/EHI.S6292 21487531 PMC3072213

[B2] Albersmeyer M. Hilge R. Schrottle A. Weiss M. Sitter T. Vielhauer V. (2012). Acute kidney injury after ingestion of rhubarb: secondary oxalate nephropathy in a patient with type 1 diabetes. *BMC Nephrol.* 13:141 10.1186/1471-2369-13-141 PMC3504561 23110375

[B3] But P. P. Tomlinson B. Lee K. L. (1996). Hepatitis related to the Chinese medicine Shou-wu-pian manufactured from *Polygonum multiflorum*. *Vet. Hum. Toxicol.* 38 280–282. 8829347

[B4] Chen L. Huang J. Y. Xue L. (2010). Effect of compound *Polygonum multiflorum* extract on Alzheimer’s disease. *J. Cent. South Univ. Med. Sci.* 36 612–615. 10.3969/j.issn.1672-7347.2010.06.012 20622335

[B5] Cho H. C. Min H. J. Ha C. Y. Kim H. J. Kim T. H. Jung W. T. (2009). Reactivation of pulmonary tuberculosis in a patient with *Polygonum multiflorum* Thunb-induced hepatitis. *Gut Liver* 3 52–56. 10.5009/gnl.2009.3.1.52 20479902 PMC2871557

[B6] Danan G. Teschke R. (2016). RUCAM in drug and herb induced liver injury: the update. *Int. J. Mol. Sci.* 17:E14 10.3390/ijms17010014 PMC4730261 26712744

[B7] Demek L. Sporni L. (1969). A study of the toxic and therapeutic effect of cardiac glycosides during their long-term and combined use. *Kardiologiia* 9 57–63. 5373726

[B8] Ding P. Zhu J. P. (2009). Clinical observation on treatment of hypertension by *Polygonum multiflorum* granules. *J. Zhejiang Coll. Tradit. Chin. Med.* 33 493–494.

[B9] Dong H. Slain D. Cheng J. Ma W. Liang W. (2014). Eighteen cases of liver injury following ingestion of *Polygonum multiflorum*. *Complement. Ther. Med* 22 70–74. 10.1016/j.ctim.2013.12.008 24559819

[B10] Fan X. Wang J. B. Xie L. H. Dong Y. S. Han G. Hu D. (2015). A new animal model for *Polygonum multiflorum* Thunb-induced liver injury in rats and its potential mechanisms. *Toxicol. Res.* 4 1085–1097. 10.1039/C5TX00054H

[B11] Kobinger W. Wenzel B. Klupp H. (1970). Relations of heart effect, toxic effect and plasma-formation in cardiac glycosides. *Arzneimittelforschung* 20 1862–1865. 5537082

[B12] Li C. Niu M. Bai Z. Zhang C. Zhao Y. Li R. (2017). Screening for main components associated with the idiosyncratic hepatotoxicity of a tonic herb, *Polygonum multiflorum*. *Front. Med.* 10.1007/s11684-017-0508-9 [Epub ahead of print]. 28315126

[B13] Li C. Y. Tu C. Gao D. Wang R. L. Zhang H. Z. Niu M. (2016). Metabolomic study on idiosyncratic liver injury induced by different extracts of *Polygonum multiflorum* in rats integrated with pattern recognition and enriched pathways analysis. *Front. Pharmacol.* 7:483 10.3389/fphar.2016.00483 PMC5156827 28018221

[B14] Li H. Wang X. Liu Y. Pan D. Wang Y. Yang N. (2016). Hepatoprotection and hepatotoxicity of Heshouwu, a Chinese medicinal herb: context of the paradoxical effect. *Food Chem. Toxicol.* 10.1016/j.fct.2016.07.035 [Epub ahead of print]. 27484243

[B15] Li X. X. Du F. Y. Liu H. X. Ji J. B. Xing J. (2015). Investigation of the active components in *Tripterygium wilfordii* leading to its acute hepatotoxicty and nephrotoxicity. *J. Ethnopharmacol.* 162 238–243. 10.1016/j.jep.2015.01.004 25582490

[B16] Lin L. Lin H. Zhang M. Ni B. Yin X. Qu C. (2015a). A novel method to analyze hepatotoxic components in *Polygonum multiflorum* using ultra-performance liquid chromatography-quadrupole time-of-flight mass spectrometry. *J. Hazard. Mater.* 299 249–259. 10.1016/j.jhazmat.2015.06.014 26135484

[B17] Lin L. Ni B. Lin H. Zhang M. Li X. Yin X. (2015b). Traditional usages, botany, phytochemistry, pharmacology and toxicology of *Polygonum multiflorum* Thunb.: a review. *J. Ethnopharmacol.* 159 158–183. 10.1016/j.jep.2014.11.009 25449462 PMC7127521

[B18] Lin L. Ni B. Lin H. Zhang M. Yan L. Qu C. (2015c). Simultaneous determination of 14 constituents of Radix polygoni multiflori from different geographical areas by liquid chromatography-tandem mass spectrometry. *Biomed. Chromatogr.* 29 1048–1055. 10.1002/bmc.3391 25450501

[B19] Lv G. P. Meng L. Z. Han D. Q. Li H. Y. Zhao J. Li S. P. (2015). Effect of sample preparation on components and liver toxicity of *Polygonum multiflorum*. *J. Pharm. Biomed. Anal.* 109 105–111. 10.1016/j.jpba.2015.02.029 25766851

[B20] Ma J. Zheng L. He Y. S. Li H. J. (2015). Hepatotoxic assessment of Polygoni Multiflori Radix extract and toxicokinetic study of stilbene glucoside and anthraquinones in rats. *J. Ethnopharmacol.* 162 61–68. 10.1016/j.jep.2014.12.045 25557036

[B21] Mazzanti G. Battinelli L. Daniele C. Mastroianni C. M. Lichtner M. Coletta S. (2004). New case of acute hepatitis following the consumption of Shou Wu Pian, a Chinese herbal product derived from *Polygonum multiflorum*. *Ann. Intern. Med.* 140:W30. 10.7326/0003-4819-140-7-200404060-00042-w3 15069011

[B22] Mengs U. Stotzem C. D. (1993). Renal toxicity of aristolochic acid in rats as an example of nephrotoxicity testing in routine toxicology. *Arch. Toxicol.* 67 307–311. 10.1007/BF01973700 8368940

[B23] Murayama M. Mori T. Bando H. Amiya T. (1991). Studies on the constituents of *Aconitum* species. IX. The pharmacological properties of pyro-type aconitine alkaloids, components of processed aconite powder ’kako-bushi-matsu’: analgesic, antiinflammatory and acute toxic activities. *J. Ethnopharmacol.* 35 159–164. 10.1016/0378-8741(91)90068-O 1809822

[B24] Ng S. W. Ching C. K. Chan A. Y. Mak T. W. (2013). Simultaneous detection of 22 toxic plant alkaloids (aconitum alkaloids, solanaceous tropane alkaloids, sophora alkaloids, strychnos alkaloids and colchicine) in human urine and herbal samples using liquid chromatography-tandem mass spectrometry. *J. Chromatogr. B Analyt. Technol. Biomed. Life Sci.* 94 63–69. 10.1016/j.jchromb.2013.10.020 24216273

[B25] Okada H. Watanabe Y. Inoue T. Kobayashi T. Kanno Y. Shiota G. (2003). Transgene-derived hepatocyte growth factor attenuates reactive renal fibrosis in aristolochic acid nephrotoxicity. *Nephrol. Dial. Transplant.* 18 2515–2523. 10.1093/ndt/gfg440 14605273

[B26] Panis B. Wong D. R. Hooymans P. M. De Smet P. A. Rosias P. P. (2005). Recurrent toxic hepatitis in a Caucasian girl related to the use of Shou-Wu-Pian, a Chinese herbal preparation. *J. Pediatr. Gastroenterol. Nutr.* 41 256–258. 10.1097/01.MPG.0000164699.41282.67 16056110

[B27] Park G. J. Mann S. P. Ngu M. C. (2001). Acute hepatitis induced by Shou-Wu-Pian, a herbal product derived from *Polygonum multiflorum*. *J. Gastroenterol. Hepatol.* 16 115–117. 10.1046/j.1440-1746.2001.02309.x 11206309

[B28] Qu K. Shen N. Y. Xu X. S. Su H. B. Wei J. C. Tai M. H. (2013). Emodin induces human T cell apoptosis in vitro by ROS-mediated endoplasmic reticulum stress and mitochondrial dysfunction. *Acta Pharmacol. Sin.* 34 1217–1228. 10.1038/aps.2013.58 23811723 PMC4003158

[B29] Teschke R. Eickhoff A. (2015). Herbal hepatotoxicity in traditional and modern medicine: actual key issues and new encouraging steps. *Front. Pharmacol.* 6:72 10.3389/fphar.2015.00072 PMC4407580 25954198

[B30] Teschke R. Larrey D. Melchart D. Danan G. (2016). Traditional Chinese Medicine (TCM) and herbal hepatotoxicity: RUCAM and the role of novel diagnostic biomarkers such as microRNAs. *Medicines* 3:18 10.3390/medicines3030018 PMC5456249 28930128

[B31] Teschke R. Schwarzenboeck A. Eickhoff A. Frenzel C. Wolff A. Schulze J. (2013). Clinical and causality assessment in herbal hepatotoxicity. *Expert Opin. Drug Saf.* 12 339–366. 10.1517/14740338.2013.774371 23458441

[B32] Teschke R. Wolff A. Frenzel C. Schulze J. (2014). Review article: herbal hepatotoxicity–an update on traditional Chinese medicine preparations. *Aliment. Pharmacol. Ther.* 40 32–50. 10.1111/apt.12798 24844799

[B33] Wang C. Wu X. Chen M. Duan W. Sun L. Yan M. (2007). Emodin induces apoptosis through caspase 3-dependent pathway in HK-2 cells. *Toxicology* 231 120–128. 10.1016/j.tox.2006.11.064 17240509

[B34] Wang J. B. Kong W. J. Wang H. J. Zhao H. P. Xiao H. Y. Dai C. M. (2011). Toxic effects caused by rhubarb (*Rheum palmatum* L.) are reversed on immature and aged rats. *J. Ethnopharmacol.* 134 216–220. 10.1016/j.jep.2010.12.008 21163343

[B35] Wang Y. Y. Li J. Wu Z. R. Zhang B. Yang H. B. Wang Q. (2017). Insights into the molecular mechanisms of *Polygonum multiflorum* Thunb-induced liver injury: a computational systems toxicology approach. *Acta Pharmacol. Sin.* 38 719–732. 10.1038/aps.2016.147 28239160 PMC5457689

[B36] Wu L. Wang B. Zhao M. Liu W. Zhang P. Shi Y. (2016). Rapid identification of officinal akebiae caulis and its toxic adulterant aristolochiae manshuriensis caulis (*Aristolochia manshuriensis*) by loop-mediated isothermal amplification. *Front Plant Sci* 7:887 10.3389/fpls.2016.00887 PMC4913086 27379153

[B37] Yang Y. Y. Wang H. S. Yang S. B. (2013). Clinical observation on treatment of nonalcoholic fatty liver disease with *Polygonum multiflorum* granules. *Chin. J. Mod. Drug Appl.* 7 63–64.

[B38] Yu J. Xie J. Mao X. J. Wang M. J. Li N. Wang J. (2011). Hepatoxicity of major constituents and extractions of Radix Polygoni Multiflori and Radix Polygoni Multiflori Praeparata. *J. Ethnopharmacol.* 137 1291–1299. 10.1016/j.jep.2011.07.055 21840387

[B39] Yuan J. B. Huang Q. Ren G. Shi M. Chen L. Yang W. L. (2014). Acute and subacute toxicity of the extract of *Aristolochiae fructus* and honey-fried *Aristolochiae fructus* in rodents. *Biol. Pharm. Bull.* 37 387–393. 10.1248/bpb.b13-00736 24369268

[B40] Zhang D. K. Li R. S. Han X. Li C. Y. Zhao Z. H. Zhang H. Z. (2016). Toxic constituents index: a toxicity-calibrated quantitative evaluation approach for the precise toxicity prediction of the hypertoxic phytomedicine-aconite. *Front. Pharmacol.* 7:164 10.3389/fphar.2016.00164 PMC4911369 27378926

[B41] Zhang L. Yan J. Liu X. Ye Z. Yang X. Meyboom R. (2012). Pharmacovigilance practice and risk control of Traditional Chinese Medicine drugs in China: current status and future perspective. *J. Ethnopharmacol.* 140 519–525. 10.1016/j.jep.2012.01.058 22374080

[B42] Zhu S. M. (2011). Clinical observation on 52 cases of pregnancy constipation treated by *Polygonum multiflorum* granules. *Zhejiang J. Tradit. Chin. Med.* 46 129–130.

[B43] Zhu Y. Niu M. Chen J. Zou Z. S. Ma Z. J. Liu S. H. (2016). Hepatobiliary and pancreatic: comparison between Chinese herbal medicine and Western medicine-induced liver injury of 1985 patients. *J. Gastroenterol. Hepatol.* 31 1476–1482. 10.1111/jgh.13323 26896664

