# Supplementary material for: A New Perspective on Liver Injury by Traditional Chinese Herbs Such As Polygonum multiflorum: The Geographical Area of Harvest As an Important Contributory Factor
Source: Front Pharmacol. 2017 Jun 20;8:349. doi: 10.3389/fphar.2017.00349 (PMC5476691; doi:10.3389/fphar.2017.00349)
Supplement: Supplementary file 1 [file Presentation_1.pdf]

Supplementary Material

A new perspective on liver injury by traditional Chinese herbs such as *Polygonum multiflorum*: the geographical area of harvest as an important contributory factor

Longfei Lin, Hui Li, Hongmei Lin, Miao Zhang, Changhai Qu, Lei Yan, Xingbin Yin, Jian Ni*

*** Correspondence:** Jian Ni: njtcm@263.net

1. Preparation of the various fractions of the PM samples

PM was extracted by boiling three times in 10 volumes of 70% ethanol and then extracted once with 10 volumes water. Each extraction was for 1.5 h. The resulting filtrates were combined and concentrated to dryness under reduced pressure to produce the **total extract**. Another PM sample was also extracted by the same methods, and the 70% ethanol extracts were combined. The solution was recovered and concentrated, then extracted five times with the same volume of dichloromethane (DCM). The DCM solutions were combined, concentrated and dried to obtain the **DCM fraction** (the content of which was mainly free anthraquinones). After extraction with DCM, a portion was separated using HPD-300 macroporous resin, and the combined water and 10% ethanol elutions were designated the **water fraction** (the content of which was mainly tannins and polysaccharides**)**; the 30% ethanol elution was designated the **30% ethanol fraction** (which was mainly composed of polyhydroxystilbenes such as TSG**)**; and the combined 70% ethanol and 95% ethanol elution fractions were considered to be the **70% ethanol fraction** (which was mainly composed of conjugated anthraquinones such as EDG**).**

2. Analysis of the main constituents of the various fractions from the different geographical areas/batches of PM

The DMSO stock solutions were diluted 100-fold with a 50% methanol–water solution prior to use in the cell assays. The diluted solutions were vortexed for 30 s and then centrifuged at 14,000 rpm for 10 min. After another centrifugation at 14,000 rpm for 10 min, the supernatants were collected for UPLC-Q-TOF/MS analysis. The analysis of the main constituents in the different PM

fractions was performed on a Waters Xevo G2 Q-TOF/MS (Waters Corp., Milford, MA, USA) in the negative detection mode. The mass spectrometric parameters were as follows: Cone gas flow rate: 50 L/h; Source temperature: 120 ℃; Capillary: 2.5 kV; Desolvation temperature: 350 ℃; Gas flow rate: 600 L/h; Full-scan range: 50 m/z to 1200 m/z. The analysis of emodin, TSG and EDG in the various fractions of PM were performed using an HPLC system coupled with a UV-Detector (SHIMADZU Corporation, Tokyo, Japan).

The contents of emodin, TSG and EDG in the five fractions of PM were calculated using external standards, and the results are summarized in **Supplementary Table 1**. These results indicate that the content of each component was relatively low in the water fraction; TSG was mainly present in the 30% ethanol fraction; emodin was mainly present in the DCM fraction; and EDG was mainly present in the 70% ethanol fraction.

Supplementary Table 1. The percentage contents of three major compounds in the five extracts.

Group	Dried extract rate (%)	TSG (%)	EDG (%)	Emodin (%)
Total part	36.67	9.65	1.00	0.22
Water part	23.33	0.54	0.07	0.005
30% ethanol part	7.33	35.55	0.89	0.024
70% ethanol part	2.35	24.27	12.68	0.17
DCM part	0.54	0.52	0.24	11.32

3. Identification of 16 potentially toxic constituents in PM

The potential toxic constituents were identified mainly through the ‘Metascope,’ which is an important function of Progenesis QI software. Metascope searched for compound identifications based not only on neutral mass and retention time but also on collisional cross-sectional area and MS/MS fragmentation data. Compounds **1, 2, 3, 4, 5, 7, 8, 13, 14, 15** and **16** were identified through Metascope. The results are shown in Table 2, and the MS/MS fragmentation data are shown in **Supplementary Figure 1**. Compounds **3** and **8** were identified as TSG and EDG, respectively, by comparison with the standards. Compounds **6**, **11** and **12** yielded the same ions at *m/z* 431.7 and 269.2, which indicated that these compounds might be derivatives of emodin. The $[M-H]^-$ ions for these compounds were 511.0556, 517.0978 and 473.1084, respectively. These compounds were characterized as emodin-*O*-hex-sulfate, emodin-*O*-(malonyl)-hex and emodin-*O*-(acetyl)-hex (Xiao

et al., 2000;Peng et al., 2013;Qiu et al., 2013) based on the literature. The $[M-H]^-$ ion of compound **10** was observed at *m/z* 559.1451 and produced ions at *m/z* 245.2, 230.4, 215.4 and 312.9, 169.5, which indicated the presence of torachrysone and galloyl moieties. Thus, compound **10** was tentatively identified as torachrysone-*O*-glucogallin.

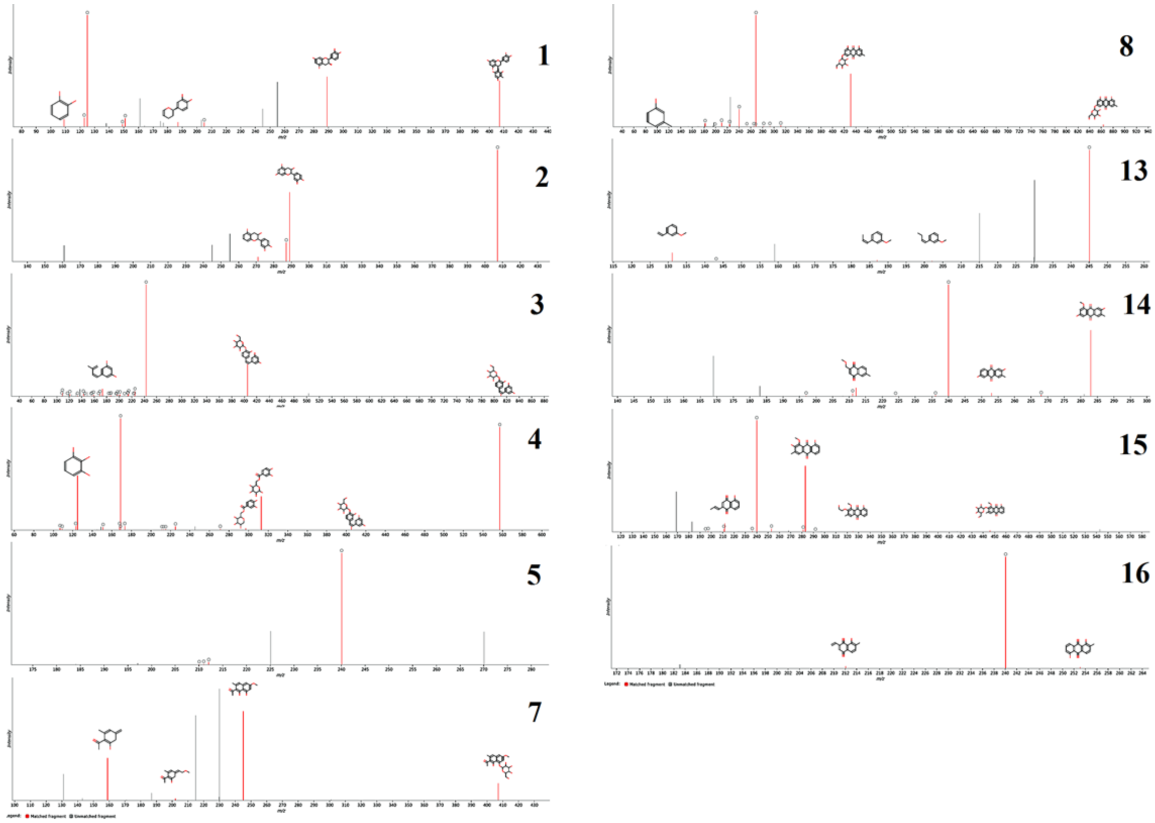

Supplementary Figure 1. The potentially toxic compounds identified through Metascope (an identification function of Progenesis QI software)

4. Correlation analysis of the content of 16 potential toxic constituents in the various fractions of PM

The correlation analysis of the content of 16 potential toxic constituents in the various fractions of PM was performed using Progenesis QI software. The TIC spectra of the various fractions of PM were collected using UPLC-Q-TOF/MS and imported into the Progenesis QI software for translation and peak picking. The 16 potentially toxic constituents were selected and marked based on retention time and *m/z*. The results showed that tetrahydroxystilbene-O-(galloyl)-hex TSG and emodin-O-hex-sulphate were positively correlated with the toxicity of the various fractions of PM, as shown in **Supplementary Figure 2.**

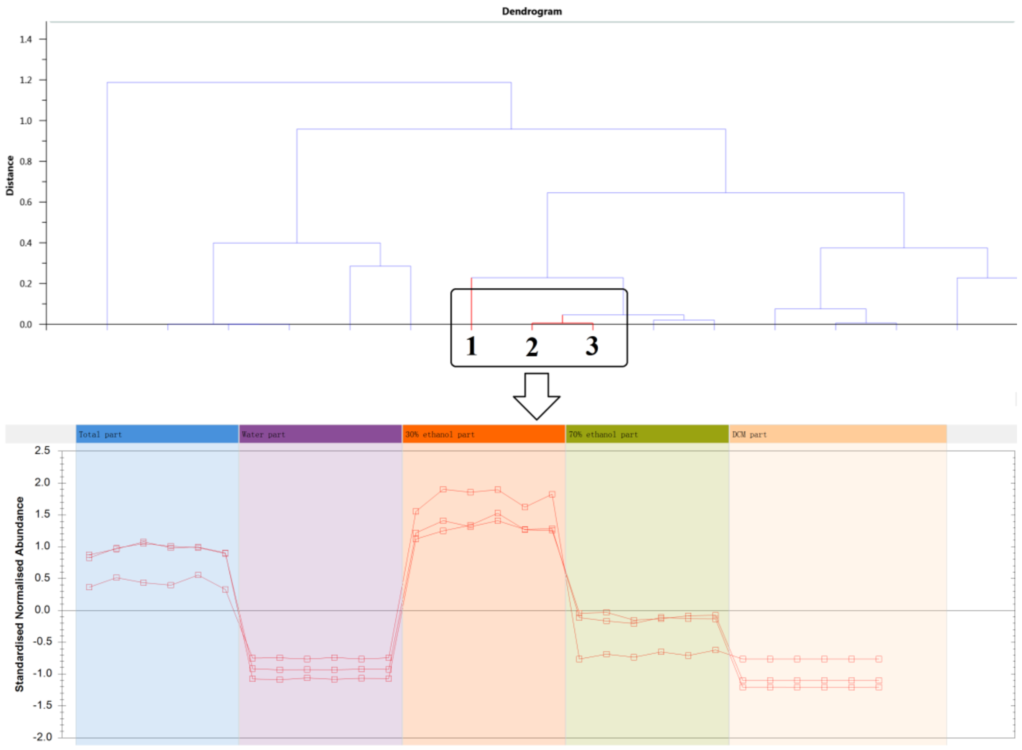

Supplementary Figure 2. The results of the correlation analysis for the content of 16 potentially toxic constituents in the various fractions of PM (1: tetrahydroxystilbene-O-(galloyl)-hex; 2: emodin-O-hex-sulphate; 3: TSG).
